# Sex‐specific telomere length and dynamics in relation to age and reproductive success in Cory’s shearwaters

**DOI:** 10.1111/mec.15399

**Published:** 2020-03-29

**Authors:** Christina Bauch, Marie Claire Gatt, José Pedro Granadeiro, Simon Verhulst, Paulo Catry

**Affiliations:** ^1^ MARE‐Marine and Environmental Sciences Centre ISPA‐Instituto Universitário Lisbon Portugal; ^2^ Groningen Institute for Evolutionary Life Sciences University of Groningen Groningen The Netherlands; ^3^ CESAM‐Centre for Environmental and Marine Studies Faculty of Science University of Lisbon Lisbon Portugal

**Keywords:** ageing, biomarker, fitness, life‐history, reproduction, survival

## Abstract

Individuals in free‐living animal populations generally differ substantially in reproductive success, lifespan and other fitness‐related traits, but the molecular mechanisms underlying this variation are poorly understood. Telomere length and dynamics are candidate traits explaining this variation, as long telomeres predict a higher survival probability and telomere loss has been shown to reflect experienced “life stress.” However, telomere dynamics among very long‐lived species are unresolved. Additionally, it is generally not well understood how telomeres relate to reproductive success or sex. We measured telomere length and dynamics in erythrocytes to assess their relationship to age, sex and reproduction in Cory's shearwaters (*Calonectris borealis*), a long‐lived seabird, in the context of a long‐term study. Adult males had on average 231 bp longer telomeres than females, independent of age. In females, telomere length changed relatively little with age, whereas male telomere length declined significantly. Telomere shortening within males from one year to the next was three times higher than the interannual shortening rate based on cross‐sectional data of males. Past long‐term reproductive success was sex‐specifically reflected in age‐corrected telomere length: males with on average high fledgling production were characterized by shorter telomeres, whereas successful females had longer telomeres, and we discuss hypotheses that may explain this contrast. In conclusion, telomere length and dynamics in relation to age and reproduction are sex‐dependent in Cory's shearwaters and these findings contribute to our understanding of what characterises individual variation in fitness.

## 1 INTRODUCTION

In wild populations, large differences exist in fitness‐related life‐history traits between individuals (e.g., Fay, Barbraud, Delord, & Weimerskirch, 2018; Hamel, Côté, Gaillard, & Festa‐Bianchet, 2009). To understand this life‐history diversity, which ultimately affects population demography and dynamics (Coulson et al., 2006; Hamel et al., 2018; Pelletier, Clutton‐Brock, Pemberton, Tuljapurkar, & Coulson, 2007), it is necessary to investigate the mechanisms that mediate it. In recent years, telomere length (TL) has emerged as a biomarker of ageing and individual state (Young, 2018). Telomeres are evolutionarily conserved DNA sequence repeats, which form the ends of chromosomes together with specific proteins and contribute to genome stability (O’Sullivan & Karlseder, 2010). TL varies considerably between individuals from very early life onwards (Sabharwal et al., 2017) due to inheritance and variation in telomere dynamics over life (Bauch, Boonekamp, Korsten, Mulder, & Verhulst, 2019; Dugdale & Richardson, 2018). Short TL is a biomarker of reduced health and survival probability in many organisms (Boonekamp, Simons, Hemerik, & Verhulst, 2013; Joeng, Song, Kong‐Joo, & Lee, 2004; Opresko & Shay, 2017; Wilbourn et al., 2018). Telomeres shorten due to incomplete replication during cell division, which can be accelerated by DNA‐ and protein‐damaging factors and attenuated or counteracted by maintenance processes (Chan & Blackburn, 2004). Telomere shortening rate has been shown to differ between individuals, for example in relation to exposure to stress, resource‐based life‐history trade‐offs or environmental conditions, and it has thus been suggested to reflect somatic costs of exposure to these challenges (Angelier, Costantini, Blévin, & Chastel, 2018; Monaghan, 2014; Young, 2018).

Meta‐analyses investigating the cross‐sectional relationship between TL and age in bird and mammal species have shown a higher telomere decline in short‐lived species (Haussmann et al., 2003; Tricola et al., 2018), which was confirmed by a meta‐analysis on the few available longitudinal studies (Sudyka, Arct, Drobniak, Gustafsson, & Cichoń, 2016). However, the pattern is inconsistent among the longest‐lived species of birds (and even less is known for wild mammals), with the cross‐sectional relationship between TL and age found to be positive (oystercatcher *Haematopus ostralegus*, Leach's storm petrel *Oceanodroma leucorhoa*: Tricola et al., 2018), insignificant when tested both longitudinally (Adélie penguin *Pygoscelis adeliae*: Beaulieu, Reichert, Le Maho, Ancel, & Criscuolo, 2011; Magellanic penguin *Spheniscus magellanicus*: Cerchiara et al., 2017) or cross‐sectionally (black‐browed albatross *Thalassarche melanophrys*: Angelier, Weimerskirch, Barbraud, & Chastel, 2019; Magellanic penguin: Cerchiara et al., 2017), or negative when including data from chicks (southern giant petrel *Macronectes giganteus*: Foote, Daunt, et al., 2011; European shag *Phalacrocorax aristotelis*, wandering albatross *Diomedea exulans*: Hall et al., 2004; northern fulmar *Fulmarus glacialis,* Tricola et al., 2018). Potential and nonmutually exclusive explanations for the diverse relationships found in these very long‐lived species are (i) telomere lengthening or (ii) telomere maintenance during adulthood and telomere shortening restricted to early life, or (iii) telomere shortening and selective disappearance of individuals with short telomeres (Haussmann & Mauck, 2008). At the population level, the observed cross‐sectional relationship can differ from the effect within individuals to the point of showing the opposite trend (van de Pol & Verhulst, 2006). This highlights the need to carry out longitudinal investigations of telomere dynamics in long‐lived species to establish within‐individual changes with age. Long‐lived species are particularly interesting for the study of telomere dynamics, as the costs of telomere erosion, or the processes of senescence it reflects, may be more likely to become apparent in species where extrinsic mortality is low.

Differences in senescence patterns or lifespan between the sexes have been found within some species (e.g., invertebrates: Jemielity et al., 2007; vertebrates: Beirne, Delahay, & Young, 2015; Steenstrup et al., 2017). A meta‐analysis in humans, for example, has shown that females, which generally live longer, have longer telomeres than males (Gardner et al., 2014). This has raised the question of whether at the cellular level TL and/or telomere shortening are involved in the mechanism underlying the sex difference in longevity, but the relationship between sex, telomeres and lifespan in other taxa is not clear (Barrett & Richardson, 2011). Differences in telomere shortening between the sexes have been explained by sex‐specific patterns of resource allocation, due to differences in requirements. For example, telomere loss has been associated with different body size of the sexes in southern giant petrels, potentially reflecting a trade‐off between growth and somatic maintenance (Foote, Daunt, et al., 2011) and has been related to different roles of the sexes during reproduction in some species (Bauch, Becker, & Verhulst, 2013; Bauch, Riechert, Verhulst, & Becker, 2016; Ryan et al., 2018), but not in others (Young et al., 2013).

The relationship between TL and reproductive success has been studied across taxa and shown to be positive in several studies (Angelier et al., 2019; Atema, 2017; Le Vaillant et al., 2015; Parolini et al., 2017; Pauliny, Wagner, Augustin, Szép, & Blomqvist, 2006), which has been interpreted to arise from  heterogeneity of individual quality in natural populations (Angelier et al., 2019). Thus, individuals of high quality potentially have enough resources to invest in both, reproduction and self‐maintenance, as opposed to lower quality individuals. However, in other studies the relationship between TL and reproductive success was negative (Bauch et al., 2013; Plot, Criscuolo, Zahn, & Georges, 2012; Ryan et al., 2018), suggesting that when high reproductive success is due to high reproductive effort this is achieved at the expense of TL (Bauch et al., 2016; Sudyka, Arct, Drobniak, Gustafsson, & Cichoń, 2019). An increased reproductive effort could lead to elevated oxidative stress or lower mitochondrial efficiency and consequently enhance telomere loss (Haussmann & Marchetto, 2010; Stier, Reichert, Criscuolo, & Bize, 2015). When tested experimentally, telomere loss reflected reproductive effort in studies that manipulated the possibility to reproduce (Heidinger et al., 2012; Kotrschal, Ilmonen, & Penn, 2007), manipulated brood size (Reichert et al., 2014; Sudyka et al., 2014), or increased stress or activity via experimental treatment with corticosterone (Schultner, Moe, Chastel, Bech, & Kitaysky, 2014), but not in studies that manipulated foraging effort or parental provisioning by increasing workload (Atema, 2017; Beaulieu et al., 2011). Better knowledge of the relationship between telomere dynamics and reproduction will increase our understanding of within‐ and between‐individual differences in reproductive success and how this varies between species.

We measured TL using telomere restriction fragment (TRF) analysis in erythrocytes of Cory's shearwaters (*Calonectris borealis*), a long‐lived seabird of the order Procellariiformes, sampled in two consecutive years. Our study individuals are part of a long‐term population study and were of known sex, (estimated) age and long‐term past reproductive success (Campioni, Granadeiro, & Catry, 2016). Our aims were (i) to test the relationship between TL and age in the population of this long‐lived seabird species, based on individuals with ages ranging from 7 to 36 years, and (ii) to assess telomere dynamics within individuals between years, to be able to disentangle patterns at the population level from within‐individual effects. To investigate the relationship between telomeres and reproduction, we analysed (iii) past average long‐term reproductive success and TL cross‐sectionally. If TL is a biomarker of individual quality, individuals with higher reproductive success would be characterized by longer telomeres. However, shorter telomeres could be a sign that higher reproductive success was achieved by higher reproductive effort, which in turn led to higher telomere loss and consequently shorter telomeres (as suggested by Bauch et al., 2013). Therefore, we tested (iv) reproductive success and simultaneous and subsequent telomere dynamics within a year (longitudinally) and (v) manipulated reproduction in a subset of individuals by removing their single‐egg clutch and related it to subsequent telomere dynamics. Thus, if reproductive effort comes at the expense of somatic maintenance, one could expect a comparatively higher telomere loss in individuals raising chicks to fledging compared to individuals that lose their egg or chick and hence undertake lower parental effort. However, if heterogeneity in phenotypic quality masks such an effect, we would expect to detect a reduced telomere loss only in an experimental set‐up, in individuals freed from parental care. (vi) Furthermore, we tested for sex differences in TL and telomere dynamics throughout the study as Cory's shearwater males and females differ in the following traits: (a) Life‐history theory predicts a resource allocation trade‐off between growth and self‐maintenance (Stearns, 1992). If this is reflected in TL, males would be expected to have shorter telomeres than females as they are the larger sex in this species (Granadeiro, 1993). (b) Males provide more parental care than females in this species (Granadeiro, Burns, & Furness, 1998), which may be reflected in higher telomere loss in males. (c) Males have lower survival rates than females (Mougin, Jouanin, & Roux, 2000a), which may be reflected in their telomeres, either by shorter TL in males or by higher telomere loss if the latter better reflects senescence.

## 2 MATERIALS AND METHODS

### 2.1 Study species

We studied Cory's shearwaters (*Calonectris borealis*) breeding on Selvagem Grande (30°09′N, 15°52′W), a 4km^2^ island nature reserve located ~300 km south of the Madeiran archipelago of Portugal. This population is subject to a long‐term study, where birds have been ringed since 1978 (with a reduced intensity only between 2000 and 2003) and reproductive success (fledging success) in ~500 nests has been monitored annually since 2004 (Campioni et al., 2016; Mougin et al., 2000a). There is no terrestrial predation on the island. Our data set contains breeding birds aged between 7 and 36 years (females: *n* = 79, mean ± *SD*: 17.49 ± 4.91 years; males: *n* = 101, mean ± *SD*: 17.14 ± 5.93 years). Fifty‐two of the 180 birds included in this study were marked with numbered metal rings as chicks for lifelong identification, and hence their exact age is known (mean age ± *SD*: 16.35 ± 8.12 years). One twenty eight birds were ringed as adults and presumed to be recruits when first captured, as breeding birds are highly philopatric (Mougin, Granadeiro, Jouanin, & Roux, 1999) and birds that occupy nests (successful and unsuccessful breeding attempts) have been identified regularly in our study area. Therefore, an age of 9 years was assigned to these birds at ringing, as 8.9 (±1.7) years is the average (±*SD*) age of recruitment in this colony for both sexes (Mougin et al., 2000a). The mean (±*SD*) age of those birds during our study was 17.7 ± 4.0 years. The sexes are dimorphic with males being on average larger in all available morphological characteristics (Granadeiro, 1993). Study birds were sexed with high accuracy (>99%) using a combination of three methods: a discriminant function based on bill measurements (Granadeiro, 1993), vocalizations (Thibault, Bregatgnolle, & Rabuñal, 1997) and a cross‐validation of the sexes of breeding partners.

The studied birds bred in individually marked artificial nest cavities in stone walls on the island plateau. Like other members of the Procellariiformes, they lay a single egg per breeding attempt and there are no replacement clutches (Warham, 1990). Birds return from their wintering areas between February and April, with males arriving on average earlier than females to secure and defend nest cavities, frequently resulting in intraspecific fights, while females attend the nest‐site less often and are absent from the colony for the period of egg formation prior to laying (Catry, Dias, Phillips, & Granadeiro, 2013; Granadeiro et al., 1998; Ramos, Monteiro, Sola, & Moniz, 1997). Egg laying in the colony occurs from late May to early June and the incubation period lasts ~54 days (Mougin, Jouanin, Roux, & Zino, 2000b). Chicks fledge after ~97 days. The parents equally share incubation, but fathers visit the nest more frequently during prelaying and chick rearing (Granadeiro et al., 1998).

### 2.2 Manipulation of reproduction

In 2017, reproduction was experimentally manipulated in 25 randomly chosen ringed breeding pairs in the study nests by removing the clutch between late June and early July, thus inducing reproduction failure and freeing birds from subsequent parental effort. Individuals of similar ages (23 males and 39 females) breeding at the same time were assigned as the control group (i.e., without manipulation).

24 males (96%) and 22 females (88%) from the 50 manipulated individuals and 20 males (87%) and 33 females (85%) from the control group were recorded and resampled in 2018.

### 2.3 Blood sample collection and telomere analysis

Adult birds were caught at their nest‐sites and blood sampled by puncturing the vena brachialis between June and July 2017 during incubation and again directly after returning from the wintering grounds between February and April 2018. Samples were first stored in 2% EDTA buffer at 4–7°C and then snap frozen in 40% glycerol buffer for permanent storage at −80°C within 4 weeks of collection. We measured terminally located TLs using TRF analysis without DNA denaturation (modified from Salomons et al., 2009). First, we removed the glycerol buffer, washed the cells and isolated DNA from 5 µl of erythrocytes using CHEF Genomic DNA Plug kit for preparation of intact, chromosome‐sized DNA (Bio‐Rad). Cells in the agarose plugs were digested overnight with Proteinase K at 50°C. Isolated DNA (half of the plug per sample) was restricted overnight simultaneously with *Hind*III (60 U), *Hinf*I (30 U) and *Msp*I (60 U) in NEB2 buffer (New England Biolabs) at 37°C. Subsequently, the restricted DNA was separated by pulsed‐field gel electrophoresis in a 0.8% agarose gel (Pulsed Field Certified Agarose, Bio‐Rad) at 14°C for 24 hr at 3.5 V/cm, initial switch time 0.5 s, final switch time 7.0 s. For size calibration, ^32^P‐labelled size markers (1‐kb DNA ladder, New England Biolabs; DNA Molecular Weight Marker XV, Roche Diagnostics) were added. Subsequently, gels were dried (gel dryer, Bio‐Rad, model 538) at room temperature and hybridized overnight at 37°C with ^32^P‐labelled oligonucleotides (5′‐CCCTAA‐3′)_4_ that bind to the single‐strand overhang of telomeres of nondenatured DNA. Unbound oligonucleotides were removed by washing the gel for 30 min at 37°C with 0.25 × saline‐sodium citrate buffer. The radioactive signal of the sample‐specific TL distribution was detected by a phosphor screen (MS, Perkin‐Elmer), exposed for ~20 hr, and visualized using a phosphor imager (Cyclone Storage Phosphor System, Perkin‐Elmer). TL per sample was calculated using imagej (version 1.38×) as described by Salomons et al. (2009). For each sample the limits for the telomere distribution were set lane‐specifically at the point of the lowest signal (i.e., background intensity), and the individual average of the TL distribution was used for further analysis. Samples of low quality that produced low signals were excluded (9%). Samples were run on 14 gels. Repeated samples of the same individuals were on the same gels, whereas sexes, ages and treatment groups were randomized over all gels. The coefficient of variation of one control sample of one randomly chosen Cory's shearwater run on all gels was 2.49%. Within‐individual repeatability for TL of five individuals sampled repeatedly in 2018 (sampling interval between 21 and 38 days) and analysed on the same gel was 89.9% (calculated following Lessels & Boag, 1987). Both the coefficient of variation and the repeatability values (as compared to other studies) indicate a high quality of the TL data.

### 2.4 Statistical analyses

We analysed TL variation using linear mixed effects models with restricted maximum‐likelihood estimates in r (version 3.5.1, R Core Team) using the packages lme4 (Bates, Mächler, Bolker, & Walker, 2015) and lmertest (Kuznetsova, Brockhoff, & Christensen, 2017). We selected models to test specific predictions in line with our study design. Model fit for linear mixed effects models was calculated as conditional *R* ^2^ using the package mumin (Bartoń, 2019).

To disentangle within‐ and between‐individual telomere shortening we used the method of within‐subject centring (van de Pol & Wright, 2009). To this end we used the mean age per individual and the deviation from the mean (delta age) per sample (taking into account the exact sampling interval in days). The “mean age” estimate in the model represents between‐individual differences, the cross‐sectional slope of the relationship between TL and age. The “delta age” estimate represents the within‐individual effect of telomere shortening, the slope based on longitudinal data. To test for differences in TL between the sexes, we included sex as a factor. As random effects we included bird ID to account for repeated TL data of the same individuals and gel ID to control for between‐gel differences. Subsequently, we added the interactions sex × mean age and sex × delta age to test for potential sex differences in the relationship between TL and age (cross‐sectional analysis: mean age) and for differences in within‐individual telomere dynamics (longitudinally: delta age). Based on these findings we then ran the model for the sexes separately. The data set for these analyses included all telomere data except for follow‐up measurements of manipulated individuals because of potential experimental effects.

To test if the slopes of between‐ (mean age) and within‐individual (delta age) telomere shortening in males were significantly different, we ran the model with mean age and exact age as two covariates. The exact age includes between‐ and within‐individual effects and the estimate for mean age in this model represents the difference between the between‐ and within‐individual effects (van de Pol & Wright, 2009). If mean age in this model is significant, slopes between and within individuals are significantly different, supporting a selective (dis‐)appearance of individuals in the population.

As an estimate of past long‐term reproductive success, we used the average number of fledglings over the past up to 13 years, which allows us to include a maximum of available data on reproduction and individuals of all ages, and estimated its association with TL in 2017 including sex as a factor and the interaction between sex and past reproductive success. Age was included as a covariate and gel ID as a random effect in the models. In a second analysis we reran the model and gave the data points of individuals different weights depending on the number of years or reproductive events each individual contributed. As a weighing factor we used the square root of the number of years (Sokal & Rohlf, 1995). Additionally, we ran the same models with average hatching success instead of fledging success. As results with and without the weighing factor were qualitatively identical, we only report the model without weighing factor.

We compared TLs of breeding partners using a linear model with male TL as the dependent variable and female TL as a covariate as well as male age to correct for telomere change with age in males. We also tested if telomere shortening from 2017 to 2018 was correlated among breeding partners using a Pearson correlation. As blood samples of partners were not necessarily taken on the same day, telomere shortening was corrected for sampling interval.

To test for a longitudinal relationship between reproductive success and TL (unmanipulated birds only), we ran a model including TL as the dependent variable, mean age, delta age and reproductive success (fledgling in 2017 yes or no) as covariates, and sex as a factor.

We investigated potential effects of experimental manipulation of reproduction on telomere shortening in a model with experiment group as a factor (coded 0 for birds whose egg was removed and one for control birds) and an interaction between experiment group and delta age. Bird ID was added as a random effect for repeated telomere data between years. For the model that included both sexes, nest ID was added as a random effect, but did not explain additional variance and hence is not reported.

## 3 RESULTS

### 3.1 Age and sex effects

**FIGURE 1:**
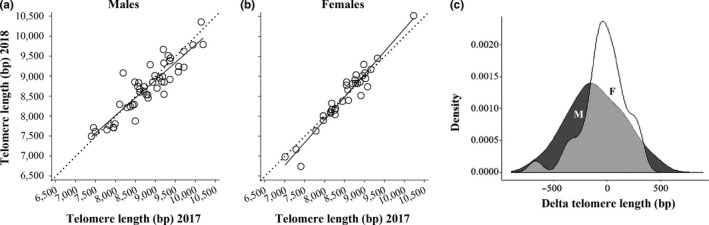
Longitudinal telomere length dynamics (unmanipulated birds only). Correlation of telomere length within (a) males and (b) females measured in 2017 and 2018. The dotted line represents *y* = *x*. Solid lines are regression lines for males (*r* = .90) and females (*r* = .97). (c) Density plot of telomere length change (delta) within individuals between the two sampling years, with negative values showing telomere shortening. Males = black, females = white. For statistics see Table 1

**FIGURE 2:**
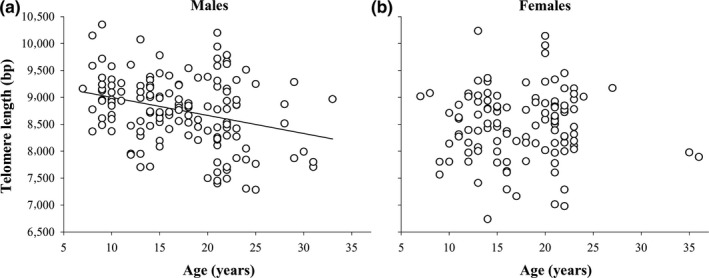
Telomere length in relation to age in (a) males and (b) females with regression lines if significant. For statistics see Table 1

TL declined significantly with age, both cross‐sectionally, comparing individuals that differ in age (“mean age,” Table 1a), and longitudinally, within individuals at different ages (“delta age,” Table 1a). TL in samples collected on the same individuals in different years was highly correlated in both sexes (Figure 1; males: *r* = .90, *n* = 47, *p* < .001; females: *r* = .97, *n* = 35, *p* < .001). Telomeres of males were on average 231 ± 94 bp longer than telomeres of females (Table 1a). Adding the interaction between “mean age” and “sex” to this model showed this to be significant (Table 1b), and we therefore repeated the analysis for the sexes separately. This revealed that among males, older individuals had shorter telomeres, and the slope of this cross‐sectional effect was −34 bp per year (“mean age,” Table 1c, Figure 2a). Longitudinal telomere shortening was approximately three times higher, at −111 bp per year (“delta age,” Table 1c). However, the difference in slope between and within individuals did not reach statistical significance (*t* = 1.33, *n* = 145 samples of 98 birds, *p* = .19). In females there was no significant telomere shortening with age, either cross‐sectionally (Table 1d, Figure 2b) or longitudinally (Table 1d, Figure 1b,c), and both effects were markedly weaker in females compared to males (Figures 1 and 2).

**TABLE 1:** Linear mixed effects models testing for telomere shortening (bp/year) in adult male and female Cory's shearwaters. Using within‐subject centring we distinguished between‐individual (mean age per bird) and within‐individual (delta age, for repeated measures the deviation from the mean) effects. (a) With sex as a fixed effect to test for sex differences in telomere length (estimate is difference in females relative to males). (b) By including interactions assessing differences in telomere dynamics between the sexes. (c) Males only. (d) Females only

Telomere length	Model terms	Estimate	*SE*	*df*	*t*	*p*
(a) Both sexes	*Intercept*	9,077.4	170.5	115.9	53.23	<.001
*n* = 258 samples/176 individuals	*Fixed effects*					
	Mean age	−18.6	8.9	168.4	−2.10	.037
	Delta age	−82.8	39.1	86.9	−2.12	.037
	Sex	−230.7	94.0	172.4	−2.46	.015
		***σ*^2^**				
	*Random effects*					
	Bird ID	0.828				
	Gel ID	0.089				
	Residual	0.084				
(b) Both sexes	*Intercept*	9,314.4	196.9	147.4	47.30	<.001
*n* = 258 samples/176 individuals	*Fixed effects*					
	Mean age	−32.6	10.6	174.8	−3.07	.002
	Delta age	−114.2	51.4	86.3	−2.22	.029
	Sex	−923.4	315.6	172.5	−2.93	.004
	Sex × mean age	40.2	17.4	170.0	2.31	.022
	Sex × delta age	68.5	79.2	86.5	0.87	.390
		***σ*^2^**				
	*Random effects*					
	Bird ID	0.825				
	Gel ID	0.089				
	Residual	0.086				
(c) Males	*Intercept*	9,329.9	190.8	78.0	48.89	<.001
*n* = 145 samples/98 individuals	*Fixed effects*					
	Mean age	−33.7	10.2	98.1	−3.31	.001
	Delta age	−111.5	57.9	51.3	−1.93	.0598
		***σ*^2^**				
	*Random effects*					
	Bird ID	0.772				
	Gel ID	0.109				
	Residual	0.119				
(d) Females	*Intercept*	8,392.1	285.4	62.7	29.41	<.001
*n* = 113 samples/78 individuals	*Fixed effects*					
	Mean age	7.97	15.4	74.3	0.52	.607
	Delta age	−46.7	47.4	35.4	−0.97	.331
		***σ*^2^**				
	*Random effects*					
	Bird ID	0.866				
	Gel ID	0.087				
	Residual	0.048				

Note

Model fit (conditional *R* ^2^): (a) *R* ^2^ = .921, (b) *R* ^2^ = .921, (c) *R* ^2^ = .892, (d) *R* ^2^ = .953.

We know the exact age of a proportion of the sampled individuals (52/180), and know an estimated age for the remainder of the birds, which could potentially bias the findings described above that are based on cross‐sectional data. We tested this by running the analyses with the subset of birds of exactly known ages only. This did not change the findings (Table S1). Therefore, we consider it reasonable to assume that data of birds that were assigned an estimated age did not bias our results (for further support see below for the results from longitudinal data that are independent of the exact ages of adult birds).

We tested for a relationship of TL or telomere loss between breeding partners. However, TLs of breeding partners were not correlated (*r* = .35, *n* = 40, *p* = .39), and neither was their telomere shortening rate from 2017 to 2018 (*r* = .055, *n* = 20, *p* = .82).

### 3.2 Natural variation in reproductive success

**FIGURE 3:**
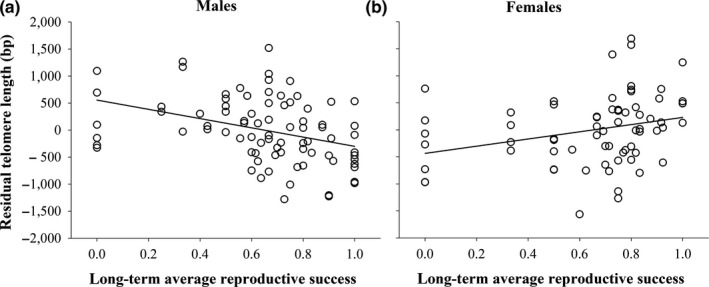
Residual telomere length (age‐corrected) in relation to average long‐term reproductive success (average fledgling production over the last up to 13 years) in (a) males and (b) females, including regression lines. For statistics see Table 2

TL was correlated with past reproductive success (average fledgling production over up to 13 years including 2017), but this pattern differed significantly between the sexes (Table 2a). More successful males were characterized by shorter age‐corrected TL (Figure 3a), while more successful females had longer age‐corrected TL (Figure 3b). Patterns in both sexes were statistically significant when tested separately (Table 2b,c) and also apparent when relating TL to past hatching success (average hatchling production over up to 13 years including 2017; Table S2). The analysis including only birds of exactly known ages supports the finding of the relationship between reproductive success and TL (Table S3). As older birds contribute data of more years to the individual average reproductive success, which could bias the results, we reran the models with weighted data of reproductive success. The results did not change our findings (compare Table S4 and Table 2).

**TABLE 2:** Linear mixed effects model testing for effects of long‐term reproductive success (average fledgling production over the past up to 13 years) on telomere length. (a) Both sexes (estimate for females relative to males), (b) males, (c) females

Telomere length	Model terms	Estimate	*SE*	*df*	*t*	*p*
(a)	*Intercept*	9,667.1	224.0	129.3	43.2	<.001
*n* = 150 birds	*Fixed effects*					
	age	−17.1	9.4	143.8	−1.82	.071
	Reprod. success	−886.7	253.7	142.1	−3.50	.001
	Sex	−1,317.6	255.2	143.1	−5.16	<.001
	Reprod. success × sex	1,656.1	360.1	141.5	4.60	<.001
		***σ*^2^**				
	*Random effects*					
	Gel ID	0.063				
	Residual	0.937				
(b)	*Intercept*	9,719.1	230.0		42.3	<.001
*n* = 82 males	*Fixed effects*					
	Age	−20.0	11.3		−1.78	.079
	Reprod. success	−898.4	248.8		−3.61	<.001
(c)	*Intercept*	8,251.3	281.9		29.27	<.001
*n* = 68 females	*Fixed effects*					
	Age	−11.4	16.2		−0.70	.484
	Reprod. success	762.9	296.8		2.57	.013

Note

Model fit (conditional *R* ^2^): (a) *R* ^2^ = .220, (b) *R* ^2^ = .202, (c) *R* ^2^ = .093.

Longitudinal analysis revealed the change in TL from 2017 to 2018 to be independent of natural variation in reproductive success in 2017 in both sexes (Table 3).

**TABLE 3:** Linear mixed effects model testing for the relationship between telomere length and loss in relation to reproductive success in 2017 in unmanipulated birds. (a) Both sexes, full model, (b) both sexes, reduced model, (c) males, and (d) females. Bird ID was included as a random effect as telomere length has been measured longitudinally

Telomere length	Model terms	Estimate	*SE*	*df*	*t*	*P*
(a)	*intercept*	9,476.5	312.6	69.2	30.3	<.001
*n* = 164 samples/82 birds	*fixed effects*					
	Mean age	−25.6	14.2	76.5	−1.80	.076
	Delta age	−223.2	110.9	78.0	−2.01	.048
	Sex	−725.3	299.2	77.0	−2.42	.018
	Fledgling	−365.5	225.2	76.0	−1.62	.109
	Fledgling × delta age	138.2	126.0	78.0	1.10	.276
	Sex × delta age	127.2	166.8	78.0	0.76	.448
	Sex × fledgling	603.5	343.2	76.1	1.76	.083
	Sex × fledgling × delta age	−72.1	190.9	78.	−0.38	.707
		***σ*^2^**				
	*Random effects*					
	Bird ID	0.900				
	Gel ID	0.022				
	Residual	0.078				
(b)	*Intercept*	9,271.9	309.9	71.6	29.9	<.001
*n* = 164 samples/82 birds	*Fixed effects*					
	Mean age	−31.9	14.4	78.6	−2.22	.030
	Delta age	−167.0	82.3	80.0	−2.03	.046
	Fledgling	−100.7	172.8	76.9	−0.58	.562
	Fledgling × delta age	104.8	94.0	80.0	1.11	.268
		***σ*^2^**				
	*Random effects*					
	Bird ID	0.887				
	Gel ID	0.040				
	Residual	0.073				
(c)	*Intercept*	9,578.8	347.7	44.0	27.55	<.001
*n* = 94 samples/47 males	*Fixed effects*					
	Mean age	−31.9	17.3	44.0	−1.85	.072
	Delta age	−223.2	125.7	45.0	−1.78	.083
	Fledgling	−360.5	218.6	44.0	−1.65	.106
	Fledgling − delta age	138.2	142.8	45.0	0.97	.338
		***σ*^2^**				
	*Random effects*					
	Bird ID	0.895				
	Residual	0.105				
(d)	*Intercept*	8,471.9	555.0	32.0	15.3	<.001
*n* = 70 samples/35 females	*Fixed effects*					
	Mean age	−12.6	24.6	32.0	−0.51	.613
	Delta age	−96.0	97.6	33.0	−0.98	.332
	Fledgling	291.6	280.4	32.0	1.04	.306
	Fledgling × delta age	66.1	112.3	33.0	0.59	.560
		***σ*^2^**				
	*Random effects*					
	Bird ID	0.956				
	Residual	0.044				

Note

Model fit (conditional *R* ^2^): (a) *R* ^2^ = .932, (b) *R* ^2^ = .932, (c) *R* ^2^ = .907, (d) *R* ^2^ = .958.

### 3.3 Manipulation of reproductive effort

Premanipulation TL, age and sampling interval did not differ between manipulated and control birds in either sex (Table 4). The reduction of parental effort achieved through experimental egg removal did not result in a reduced telomere loss of manipulated birds compared to controls (sexes combined, Table 5a). In males, there was a trend in the opposite direction, with manipulated males losing more telomere base pairs than control males (Table 5b). In females, telomere attrition was not affected by the egg removal experiment (Table 5c).

**TABLE 4:** Descriptive information (mean ± *SD*) on the individuals sampled in 2017 according to their sex and status with respect to reproduction in 2017. “Additional” birds are unmanipulated but not part of the control group. Sampling interval refers to the time elapsed between the samples taken in 2017 and 2018

	Manipulated		Control		Additional	
	Males	Females	Males	Females	Males	Females
*n*	23	24	21	38	59	17
Age (years)	17.9 ± 5.1	15.6 ± 5.3	17.2 ± 4.3	17.2 ± 4.9	15.9 ± 6.7	18.5 ± 4.2
TL (bp)	8,910 ± 526	8,452 ± 611	8,679 ± 469	8,597 ± 496	8,794 ± 732	8,656 ± 976
Sampling interval (days)	263 ± 10	253 ± 9	263 ± 14	263 ± 11	269 ± 10	264 ± 15

**TABLE 5:** Linear mixed effects model testing for effects of experimentally manipulated reproductive effort on telomere dynamics. (a) Both sexes (estimate for females relative to males), (b) males, (c) females. Bird ID was included as a random effect as telomere length has been measured longitudinally

Telomere length	Model terms	Estimate	*SE*	*df*	*t*	*p*
(a)	*Intercept*	8,674.5	256.8	69.0	33.8	<.001
*n* = 148 samples/74 birds	*Fixed effects*					
	Mean age	1.5	11.8	69.0	0.13	.901
	Delta age	−255.4	90.4	70.0	−2.83	.006
	Sex	−312.3	179.1	69.0	−1.74	.086
	Experiment group	−26.8	181.1	69.0	−0.15	.883
	Experiment group × delta age	229.5	128.3	70.0	1.79	.078
	Sex × delta age	241.7	127.3	70.0	1.90	.062
	Sex × experiment group	108.5	242.7	69.0	0.45	.656
	Sex × experiment × delta age	−293.7	173.3	70.0	−1.70	.095
		***σ*^2^**				
	*Random effects*					
	Bird ID	0.876				
	Residual	0.124				
(b)	*Intercept*	8,966.5	333.9	29.0	26.86	<.001
*n* = 64 samples/32 males	*Fixed effects*					
	Mean age	−14.0	16.6	29.0	−0.84	.407
	Delta age	−255.5	89.0	30.0	−2.87	.007
	Experiment group	−38.5	162.1	29.0	−0.24	.814
	Experiment group × delta age	229.5	126.3	30.0	1.82	.079
		***σ*^2^**				
	*Random effects*					
	Bird ID	0.852				
	Residual	0.148				
(c)	*Intercept*	8,195.8	291.0	39.0	28.17	<.001
*n* = 84 samples/42 females	*Fixed effects*					
	Mean age	11.9	16.4	39.0	0.73	.471
	Delta age	−13.7	90.7	40.0	−0.15	.880
	Experiment group	64.8	172.7	39.0	0.38	.709
	Experiment group × delta age	−64.2	117.9	40.0	−0.55	.589
		***σ*^2^**				
	*Random effects*					
	Bird ID	0.890				
	Residual	0.111				

Note

Model fit (conditional *R* ^2^): (a) *R* ^2^ = .884, (b) *R* ^2^ = .857, (c) *R* ^2^ = .891.

## 4 DISCUSSION

It is a general observation that vertebrate TL declines with age (Haussmann et al., 2003; Tricola et al., 2018), but whether this also holds true in very long‐lived species of birds—among them Procellariifomes, an order of pelagic seabirds—has not previously been confirmed within individuals. Hence our results, based on high‐precision TL data, provide the first evidence of telomere shortening in a Procellariiform during adulthood, both cross‐sectionally and longitudinally (i.e., within individuals). Furthermore, we show that TL is sex‐specifically associated with past reproductive success, positively in females and negatively in males.

### 4.1 Age and sex effects

While there was a significant decline in TL in our data set when the sexes were pooled, further analysis showed this to differ between the sexes, with TL declining faster with age in males (cross‐sectionally and longitudinally) compared to females, in which the observed decline was not statistically significant. The extent to which telomere shortening rates differ between the sexes and how and why such differences arise is not well known (Barrett & Richardson, 2011). It may be that females are generally better at maintaining their telomeres; for example, sex hormones may play a role by promoting antioxidant defence and/or telomerase activity (Aviv, 2002). Another and not mutually exclusive possibility is that the sex difference in shortening rate may reflect the division of labour during the breeding period, with males doing a larger share of nest defence and parental care in Cory's shearwaters (Granadeiro et al., 1998). Stress or higher activity during reproduction, as induced or reflected by higher corticosterone levels, have been shown to relate to higher telomere shortening (Angelier et al., 2018; Schultner et al., 2014), potentially via increased oxidative stress levels or inefficient mitochondrial efficiency (Haussmann & Marchetto, 2010; Stier et al., 2015). Furthermore, a stronger competition for reproduction in males, as suggested by frequently observed serious fights for nesting sites in our study population, could lead to higher investment in reproduction at the cost of a lower investment in self‐maintenance (reflected in higher telomere loss) and the observed lower survival probability in males (Mougin et al., 2000a). Higher rates of senescence in the sex under higher competition for reproduction has also been found in European badgers (*Meles meles*; Beirne et al., 2015). Telomeres of adult males shortened about three times faster within individuals, compared to the cross‐sectional age effect. The difference between the slopes did not reach statistical significance, which may in part be due to the fact that the within‐individual estimate was based on age differences of < 1 year. However, the difference was in the expected direction (telomere loss: within > between), in line with males with shorter telomeres being more likely to disappear from the breeding population (i.e., die), as in other studies on wild vertebrates (Wilbourn et al., 2018).

Males had on average longer telomeres than females (controlling for age), while telomere shortening was faster in adult males. This means that male TL must already be longer than female TL early in life, when breeding for the first time. Whether this sex difference is already present in the zygote, arises between the zygote stage and first breeding due to differential telomere attrition rates, or is due to sex‐specific differential TL‐dependent selection of breeding birds remains to be investigated. As females are the smaller sex in Cory's shearwaters, shorter telomeres in females cannot be explained by a trade‐off between growth and telomere maintenance as suggested for southern giant petrels, where the larger sex, males, had shorter telomeres (Foote, Daunt, et al., 2011). In lesser black‐backed gulls (*Larus fuscus*) males tended to have longer telomeres than females as hatchlings (Foote, Gault, Nasir, & Monaghan, 2011), providing support for the existence of sex differences already early in life. On the other hand, results from a study on thick‐billed murres (*Uria lomvia*) support that sex differences arise during life due to differences in telomere shortening, as TL did not differ early in life (Young et al., 2013). In humans, females have longer telomeres than males. This sex difference in TL is already present at birth and persists over life (Factor‐Litvak et al., 2016). Nevertheless, telomere dynamics differed between the sexes. Telomere shortening in females was related to the reproductive period, in that the rate of telomere loss slowed down after menopause (and thus at older ages), while in males telomere shortening tended to increase with age (Dalgård et al., 2015).

Sex differences in lifespan are known to occur in many species (e.g., Liker & Szekely, 2005), but the underlying mechanisms are not well understood (Austad & Fischer, 2016). Given that survival is associated with TL in many species, the question has been raised as to whether sex differences in TL and/or telomere shortening contribute to sex differences in lifespan (e.g., Barrett & Richardson, 2011). Our results are interesting in this respect, because in Cory's shearwaters the females enjoy a higher survival rate (Mougin et al., 2000a), while they have shorter telomeres, which is in contrast to what would be expected if TL contributed to the sex difference in lifespan. On the other hand, telomere shortening was faster in the shorter‐lived sex (males) in both Cory's shearwaters (this study) and in humans, at least later in life (Dalgård et al., 2015). These comparisons suggest telomere shortening rate to be more relevant for explaining sex differences in lifespan than absolute TL, as also found in a comparative analysis between species (Tricola et al., 2018), but more studies are needed before more definitive conclusions can be drawn with respect to sex differences in telomere dynamics.

### 4.2 Telomere length and reproductive success

Given that longer age‐corrected telomeres are usually assumed to reflect higher phenotypic quality, because of the positive association with health and survival, one could expect to also find a positive association between TL and reproductive success. While this was confirmed in some wild populations (Angelier et al., 2019; Atema, 2017; Le Vaillant et al., 2015; Pauliny et al., 2006), this pattern is not universal. For example, in another seabird species, the common tern (*Sterna hirundo*), more successful individuals had shorter telomeres than less successful individuals (Bauch et al., 2013; Bichet et al., 2020). The pattern in Cory's shearwaters is clearly more complex, with past reproductive success (average fledgling production over up to 13 years) being sex‐specifically reflected in TL, with on average more successfully reproducing males being characterized by shorter telomeres, whereas the opposite was true for females. This pattern was already apparent in the hatching success (average hatchling production over up to 13 years) that birds achieved over the years.

A possible scenario that explains the sex differences in TL is that there is a compensation of parental provisioning between the sexes (Wright & Cuthill, 1989). Thus, if females are paired with males that provision their chick at a high rate, resulting in high reproductive success, the females themselves could in turn have to work less hard, resulting in slower telomere shortening and, hence, longer age‐dependent telomeres in females. Alternatively, females with longer telomeres may obtain more successful males than females with shorter telomeres. However, neither TLs of breeding partners nor their telomere dynamics were correlated. Thus, our data did not support either of these two hypotheses, but the statistical power for these tests was limited and a larger data set is required to test these predictions with sufficient power. We consider it likely that heterogeneity in phenotypic quality is expressed as a positive covariance between TL and reproductive success in females, in line with findings in black‐browed albatrosses (Angelier et al., 2019). In this species, there was a positive association between TL and reproductive success in both sexes, consistent with chick provisioning rates being similar in the sexes (Huin, Prince, & Briggs, 2000). A stronger intrasexual reproductive competition in males than females, as mentioned previously, could lead to a negative relationship between long‐term reproductive success and TL in Cory's shearwater males. That sex differences in telomere selection exist in the wild has also been found in sand lizards (*Lacerta agilis*; Olsson et al., 2011).

### 4.3 Telomere dynamics and reproductive effort/reproductive success

TL at any point in life is the result of TL at the start of life and subsequent telomere dynamics. If a trade‐off between investment in reproduction and self‐maintenance is reflected in telomere shortening, sex‐dependent reproductive effort may have contributed to the observed sex differences in TL. Cross‐sectional data cannot resolve this question and we therefore examined longitudinal associations between TL and reproductive success. For example, common terns with higher reproductive output showed higher telomere loss, with the exception of the most successful individuals (Bauch et al., 2013). We did not detect such a longitudinal effect in our current data set in either sex, but statistical power was modest. Due to heterogeneity in individual quality, reproductive success (longer parental care) is a rather crude measure of reproductive effort. Experimental manipulations of reproductive effort could shed light on the way the observed associations between reproductive success and TL have arisen. To this end, we removed the single egg from pairs, to test experimentally for an effect of reproductive effort on telomere dynamics. The experimental reduction of parental effort in 2017 did not significantly affect telomere dynamics in either sex.

The absence of a longitudinal effect of reproductive success (natural or experimental) on TL may be explained by the relatively short sampling interval for a long‐lived species like Cory's shearwater, where slow telomere attrition can be expected (as shown in an interspecies comparison, Sudyka et al., 2016). Alternatively, only an experimental increase in reproductive effort may have had an effect on telomere dynamics, as shown in zebra finches (*Taeniopygia guttata*; Reichert et al., 2014) or as shown more generally with an effect on survival across species (Santos & Nakagawa, 2012). In our study species, however, a brood size increase would have been outside the natural range and thus an unnatural increase of reproductive effort as Cory's shearwater lay only one egg per breeding attempt. Furthermore, such an effect on telomere dynamics may only become apparent under less favourable environmental conditions, as shown for example in chicks of a different Procellariiform species (European storm petrel *Hydrobates pelagicus*) in differing years (Watson, Bolton, & Monaghan, 2015) or adult thick‐billed murres in differing colonies (Young et al., 2013). Nevertheless, and along with the sex‐specific TL‐dependent selection hypothesis, we consider that reproductive effort could have mediated the negative relationship between TL and long‐term reproductive success in males.

In conclusion, we show clear associations between TL and life histories of Cory's shearwater and these associations differed in direction between the sexes. Telomeres thereby provide a window into the physiological and molecular causes of individual and sex‐specific variation in ageing and reproductive success.

## AUTHOR CONTRIBUTIONS

P.C., J.P.G. and S.V. planned the project, M.C.G. performed the manipulation and collected samples and data in 2017 and in 2018 together with C.B., C.B. analysed telomeres, performed data analyses, and wrote the manuscript together with S.V.; all authors commented on the manuscript.

## Supporting information

Table S1‐S4

## Data Availability

The data that support the findings of this study are available from the Dryad Digital Repository: https://doi.org/10.5061/dryad.j0zpc869z (Bauch, Gatt, Granadeiro, Verhulst, & Catry, 2020).
