# Supplementary material for: Sex‐specific telomere length and dynamics in relation to age and reproductive success in Cory’s shearwaters
Source: Mol Ecol. 2020 Mar 29;29(7):1344–57. doi: 10.1111/mec.15399 (PMC7216837; doi:10.1111/mec.15399)
Supplement: Supplementary file 1 — Table S1‐S4 [file MEC-29-1344-s001.pdf]

MOLECULAR ECOLOGY

Supplemental Information for:

Sex-specific telomere length and dynamics in relation to age and reproductive success in Cory's Shearwaters

Christina Bauch, Marie Claire Gatt, José Pedro Granadeiro
& Simon Verhulst, Paulo Catry

Table of Contents:

**Table S1**<br>Telomere length in relation to exact age (cross-sectional analysis)	Page 2
**Table S2**<br>Telomere length in relation to average hatchling production	Page 3
**Table S3**<br>Telomere length in relation to average fledgling production in birds of exactly known ages<br>**Table S4**<br>Telomere length in relation to average fledgling production with a weighing factor to account for individual data contribution	Page 4

MOLECULAR ECOLOGY

Table S1. Linear mixed effects models testing for (A) sex differences in telomere length, and the cross-sectional relationship between age and telomere length (bp/year) in adult Cory's Shearwaters with exactly known ages: (B) males, (C) females.

telomere length	model terms	estimate	std. error	df	*t*	*p*
(A) both sexes	*intercept*	9298.9	215.4	39.7	43.17	<0.001
n=80 samples /	*fixed effects:*					
52 individuals	age	-35.1	11.3	53.84	-3.12	0.003
	sex	-452.8	290.9	46.5	-1.56	0.126
		σ^2				
	random effects:					
	bird ID	0.723				
	gel ID	0.191				
	residual	0.086				
(B) males	*intercept*	9333.9	205.3	49.4	45.47	<0.001
n=71 samples /	*fixed effects:*					
47 individuals	age	-37.5	11.5	50.3	-3.27	0.002
		σ^2				
	random effects:					
	bird ID	0.912				
	residual	0.088				
(C) females	*intercept*	8679.6	885.5	3.4	9.80	0.001
n= 9 samples /	*fixed effects:*					
5 individuals	age	-20.57	41.4	3.4	-0.50	0.649
		σ^2				
	random effects:					
	bird ID	0.939				
	residual	0.061				

MOLECULAR ECOLOGY

Table S2. Linear mixed effects model testing for effects of long-term hatching success (average hatchling production over the past up to 13 years) on telomere length. **(A)** Both sexes (estimate for females relative to males), **(B)** males, **(C)** females.

telomere length	model terms	estimate	std. error	df	*t*	*p*
(A)	*intercept*	9611.0	237.5	135.1	40.5	<0.001
n=150 birds	*fixed effects:*					
	age	-18.5	9.7	144.3	-1.91	0.058
	hatching success	-683.1	262.0	138.9	-2.61	0.010
	sex	-1303.9	278.5	142.8	-4.68	<0.001
	hatching success * sex	1501.1	364.5	141.1	4.12	<0.001
		σ^2				
	random effects:					
	gel ID	0.069				
	residual	0.931				
(B)	*intercept*	9645.5	245.1		39.36	<0.001
n=82 males	*fixed effects:*					
	age	-20.3	11.8		-1.71	0.091
	hatching success	-697.1	265.8		-2.62	0.011
(C)	*intercept*	8241.9	279.6		29.48	<0.001
n=68 females	*fixed effects:*					
	age	-14.9	16.5		-0.90	0.369
	hatching success	813.6	297.6		2.73	0.008

Model fit (conditional R^2): (A) R^2=0.204, (B) R^2=0.142, (C) R^2=0.101

MOLECULAR ECOLOGY

Table S3. Linear mixed effects model testing for effects of long-term reproductive success (average fledgling production over the past up to 13 years) on telomere length in male Cory's shearwater with exactly known ages. (Available sample size for females too small for a meaningful statistical result).

telomere length	model terms	estimate	std. error	*t*	*p*
	intercept	9596.8	261.8	36.66	<0.001
n=36 males	*fixed effects:*				
	age	-25.5	12.4	-2.05	0.048
	reprod. success	-670.1	284.3	-2.36	0.025

Table S4. Linear mixed effects model testing for effects of long-term reproductive success (average fledgling production over the past up to 13 years with a weighing factor (square root of number of reproductive events) to account for individual contribution) on telomere length. **(A)** Both sexes (estimate for females relative to males), **(B)** males, **(C)** females.

telomere length	model terms	estimate	std. error	df	*t*	*p*
(A)	*intercept*	9816.4	266.0	137.2	36.9	<0.001
n=150 birds	*fixed effects:*					
	age	-19.1	10.3	144.0	-1.86	0.065
	reprod. success	-1043.0	304.3	138.4	-3.43	<0.001
	sex	-1428.6	301.3	138.6	-4.74	<0.001
	reprod. success * sex	1825.4	418.1	137.7	4.37	<0.001
		σ^2				
	random effects:					
	gel ID	0.040				
	residual	0.960				
(B)	*intercept*	9839.2	275.2		35.8	<0.001
n=82 males	*fixed effects:*					
	age	-20.8	12.5		-1.66	0.100
	reprod. success	-1044.9	304.1		-3.44	<0.001
(C)	*intercept*	8306.0	335.5		24.76	<0.001
n=68 females	*fixed effects:*					
	age	-14.5	17.1		-0.85	0.401
	reprod. success	771.0	334.0		2.31	0.024

Model fit (conditional R^2): (A) R^2=0.122, (B) R^2=0.104, (C) R^2=0.035
